# Prognostic factors for survival in patients with advanced oesophageal cancer treated with cisplatin-based combination chemotherapy

**DOI:** 10.1038/sj.bjc.6601364

**Published:** 2003-11-25

**Authors:** M B Polee, W C J Hop, T C Kok, F A L M Eskens, M E L van der Burg, T A W Splinter, P D Siersema, H W Tilanus, G Stoter, A van der Gaast

**Affiliations:** 1 Departments of Medical Oncology, Erasmus Medical Center, Rotterdam, The Netherlands; 2 Departments of Epidemiology & Biostatistics, Erasmus Medical Center, Rotterdam, The Netherlands; 3 Departments of Gastroenterology and Hepatology, Erasmus Medical Center, Rotterdam, The Netherlands; 4 Department of Surgery, Erasmus Medical Center, Rotterdam, The Netherlands

**Keywords:** prognosis, survival, oesophagus, cancer, chemotherapy, cisplatin

## Abstract

The objective of this study was to identify prognostic factors for survival in patients with advanced oesophageal cancer, who are treated with cisplatin-based combination chemotherapy. We analysed the baseline characteristics of 350 patients who were treated in six consecutive prospective trials with one of the following regimens: cisplatin/etoposide, cisplatin/etoposide/5-fluorouracil, cisplatin/paclitaxel (weekly) and cisplatin/paclitaxel (biweekly). Predictive factors in univariate analyses were further evaluated using multivariate analysis (Cox regression). The median survival of all patients was 9 months. The 1, 2 and 5-year survival rates were 33, 12 and 4%, respectively. The main prognostic factors were found to be WHO performance status (0 or 1 *vs* 2), lactate dehydrogenase (normal *vs* elevated), extent of disease (limited disease defined as locoregional irresectable disease or lymph node metastases confined to either the supraclavicular or celiac region *vs* extensively disseminated disease) in addition to the type of treatment (weekly or biweekly cisplatin/paclitaxel regimen *vs* 4-weekly cisplatin/etoposide with or without 5-fluorouracil). Although weight loss, liver metastases and alkaline phosphatase were significant prognostic factors in univariate analyses, these factors lost their significance in multivariate analyses. The median survival for patients without any risk factors was 12 months, compared to only 4 months in patients with WHO 2 plus elevated LDH and extensive disease. The performance status, extent of disease, LDH and the addition of paclitaxel to cisplatin are independent prognostic factors in patients with advanced oesophageal cancer, who are treated with cisplatin-based combination chemotherapy.

The outlook for patients with oesophageal cancer is poor. Many patients who present with symptoms of oesophageal obstruction already have locally advanced or metastatic disease. The 5-year survival rate following surgery in patients thought to have localised disease is only 20% (Muller *et al*, 1990; Millikan *et al*, 1995). Most patients with oesophageal cancer need palliative treatment for local recurrence and/or metastases at some stage of the disease. Palliative surgery to relieve dysphagia carries a high morbidity and the median survival following surgery in these patients is 5–8 months. Therefore, palliative surgery has been replaced by less aggressive treatments. Intraluminal radiotherapy, intubation with self-expanding metal stents and laser therapy are all effective in palliation of dysphagia, and are associated with less morbidity than surgery (Siersema *et al*, 1998). The median survival after these types of noninvasive palliative treatment is only 3–6 months, comparable to the survival of patients with untreated advanced oesophageal cancer (Low and Pagliero, 1992; Knyrim *et al*, 1993). Despite the palliation of dysphagia, the quality of life rapidly deteriorates in most patients, due to disease-related symptoms such as pain, fatigue, appetite loss and constipation (Blazeby *et al*, 1995, 2000).

Chemotherapy may offer palliation and/or prolongation of survival in patients with advanced oesophageal cancer. Combination chemotherapy, usually cisplatin based, has been evaluated in several phase II studies and response rates of 25–40% have been reported (Enzinger *et al*, 1999). However, the impact of chemotherapy on the survival and quality of life is unknown due to a lack of randomised phase III studies comparing chemotherapy to supportive care alone. Furthermore, it is unclear which patients will benefit from palliative chemotherapy and whether potential benefits outweigh the toxicities caused by such treatment. In one of the few randomised trials that have been performed in patients with metastatic disease, the combination of cisplatin and 5-fluorouracil was more effective than cisplatin alone, but at the cost of severe toxicity (Bleiberg *et al*, 1997). In recent phase II studies, response rates after treatment with cisplatin combined with either paclitaxel (Ilson *et al*, 2000) or irinotecan (Ilson *et al*, 1999) seemed to be higher than after treatment with cisplatin and 5-fluoroura-cil; randomised studies, however, have not been performed thus far.

The outcome of chemotherapy and the prognosis of patients with oesophageal cancer most probably depend on both patient and disease characteristics. Knowledge of these factors may be useful for patient and treatment selection. In this study, we analysed a number of putative prognostic factors in patients who were treated in six consecutive prospective clinical trials with cisplatin-based chemotherapy.

## PATIENTS AND METHODS

### Patient population

Patients were entered in six consecutive prospective studies with cisplatin-based chemotherapy. The details of these studies are listed in Table 1

**Table 1:** Studies characteristics

**Study**	**Design (phase)**	**Tumor type**	**Patients no.**	**Chemotherapy**		**Schedule**	**Reference**
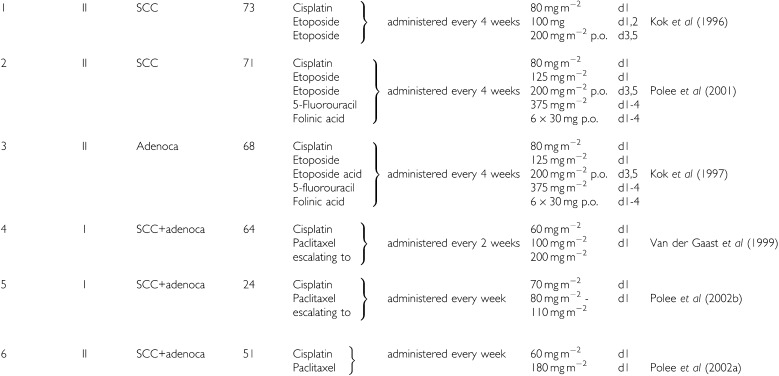							

SCC=squamous cell carcinoma.

. All patients had either squamous cell carcinoma or adenocarcinoma of the oesophagus or oesophago-gastric junction. Eligibility criteria for all these trials included histologically proven metastatic or unresectable oesophageal cancer, a life expectancy of more than 12 weeks; age ⩾18 years; WHO performance status 0–2; informed consent; adequate haematological, renal and hepatic functions defined as: granulocytes ⩾1.5 × 10^9^ l^−1^, platelets ⩾100 × 10^9^ l^−1^, total bilirubin ⩽1.5 × upper normal limit and creatinine ⩽120 *μ*mol l^−1^. Prior radiotherapy was allowed if not involving more than 30% of the bone marrow. Prior chemotherapy was not allowed. Patients had no history of other malignancies except for nonmelanomatous skin cancer or a cured malignancy more than 5 years prior to enrollment. Further exclusion criteria were: pre-existing neurotoxicity greater than common toxicity criteria (CTC) grade 1, active infection or other serious underlying medical condition which would impair the ability of the patient to receive the planned treatment, inadequate calorie and fluid intake and mental disorders not permitting adequate informed consent.

Pretreatment evaluations consisted of a complete medical history, physical examination, complete blood cell count and serum biochemistry, computerised tomography (CT) scan of the chest and upper abdomen and ultrasonography of the supraclavicular nodes when appropriate. Patients with the primary tumour *in situ* were also evaluated by endoscopy. The response to chemotherapy was evaluated by CT scan, and by ultrasonography and endoscopy when appropriate. In patients with measurable or evaluable disease, response was evaluated using WHO response criteria (WHO, 1979).

### Statistical methods

Pretreatment characteristics that were analysed for prognostic significance were age, sex, performance status, weight loss (<5, 5–10, >10%), time from diagnosis to the start of chemotherapy, histology (adenocarcinoma or squamous cell carcinoma), tumour grade, haemoglobin, alkaline phosphatase, lactate dehydrogenase (LDH), treatment with a dose-dense combination of cisplatin and paclitaxel (studies 4–6) *vs* a combination of cisplatin, etoposide with or without 5-fluorouracil (study 1,2,3), extent of disease and response to treatment.

Patients with locally irresectable disease without metastases were categorised as having locoregional disease, patients with lymph node metastases confined to either the celiac or supraclavicular region as having limited disseminated disease and patients with distant metastases or lymph node metastases in both celiac and supraclavicular lymph nodes as having extensively disseminated disease.

Statistical analysis was performed using the SPSS software (SPSS inc, Chicago, IL, USA). Survival was defined as the time elapsing from the start of chemotherapy to death or to the date of last follow-up. All survival data had been updated to August 2001. Survival curves according to the putative prognostic factors were drawn using the method of Kaplan and Meier (1958), and were compared with the log-rank test (Mantel, 1966). The factors that were univariately significantly related to prognosis were further evaluated in multivariate analyses. The Cox proportional hazards model (Cox regression) was used with backward elimination to find the most important independent prognostic factors (Cox, 1972). *P*=0.05 (two-sided) was considered the limit of significance. To circumvent difficulties with respect to the time bias (Anderson *et al*, 1983), the association between tumour response (partial or complete) and survival was assessed only in patients who survived 4 months after the start of treatment. *P*=0.05 (two-sided) was considered the limit of significance.

## RESULTS

A total of 351 patients were analysed. All patients received at least one course of cisplatin-based combination chemotherapy. One patient had no follow-up and was excluded from further analysis. The characteristics of the remaining 350 patients are listed in Table 2

**Table 2:** Patient characteristics (*n*=350)

**Characteristic**	**No. of patients**	**(%)**
*Sex*		
Male	278	79
Female	72	21
		
*Age (years)*		
Median	56	
Range	28–78	
		
*WHO performance status*		
0	66	19
1	219	63
2	63	18
		
*Weight loss* (%)		
<5	121	35
5–10	91	26
>10	131	37
Unknown	7	2
		
*Histology*		
Adenocarcinoma	145	41
Squamous cell carcinoma	199	57
Undifferentiated carcinoma	6	2
		
*Extent of disease*		
Locally advanced/unresectable	42	12
Limited disseminated disease^a^	138	39
Extensive disseminated disease^b^	170	49

a Lymph node metastasis confined to either the celiac or supraclavicular region.

b Distant metastasis or lymph node metastasis in both celiac and supraclavicular lymph nodes.

. Patients included in the database were all registered before 30 June 1999 and the date of reference for the survival analysis was 31 August 2001. At the time of this analysis, 27 patients were alive. The median survival of all patients was 9 months (95% confidence interval 8–10 months). The 1-, 2- and 5-year survival rates were 33, 12 and 4%, respectively.

### Univariate analysis

Table 3

**Table 3:** Univariate survival analysis

**Characteristic**	**Categories**	**Patients(no)**	**Survival median (months)**	**1-year (%)**	**Logrank *P***
Age (years)	<60	215	9	29	0.235
	Δ60	135	11	39	
					
Gender	Female	72	11	42	0.390
	Male	278	9	30	
					
Performance status (WHO)	0	66	12	45	<0.001^a^
	1	219	10	35	
	2	63	5	13	
					
Weight loss (%)	Ω 5	121	12	39	0.006^a^
	5–10	91	9	32	
	Δ 10	131	9	29	
					
Tumor type	SCC	199	9	32	0.550
	Adenoca	145	10	32	
					
Tumor differentiation^b^	Well/moderate	119	10	43	0.102
	Poor	131	9	28	
					
Liver metastasis	No	269	10	37	<0.001
	Yes	81	7	21	
					
No. of metastatic sites	0	42	10	36	0.02^a^
	1	190	10	34	
	>1	118	7	31	
					
Extent of disease	Locoregional	42	10	36	< 0.001^c^
	Limited dissem.	138	12	41	< 0.001^c^
	Extensive dissem.	170	7	26	
					
Interval between diagnosis and start treatment	<6 months	295	10	34	0.65
	>6 months	55	8	27	
					
Haemoglobin	Normal	228	10	36	0.253
	Abnormal	122	9	28	
					
Alkaline phosphatase	Normal	246	10	37	0.001
	Elevated	104	7	24	
					
LDH	Normal	296	11	46	<0.001
	Elevated	54	8	16	
					
Paclitaxel	Yes	139	10	40	0.002
	No	211	9	29	

a Trend test,

b tumour differentiation was unknown in 100 patients,

c *vs* extended dissemination.

summarises the results of the univariate survival analyses. Significant variables related to survival were: performance status, weight loss, LDH, alkaline phosphatase, liver metastases, number of metastatic sites, extent of disease and treatment with a dose-dense cisplatin and paclitaxel chemotherapy regimen. Patients with locoregional irresectable disease and patients with limited disseminated disease had a significantly better survival than patients with extensively disseminated disease.

### Multivariate analysis

Multivariate analysis showed that good performance score, limited disseminated disease, normal LDH and treatment with a dose-dense schedule of cisplatin and paclitaxel were independent prognostic factors (Table 4

**Table 4:** Cox multivariate regression model

**Characteristic**	**RDR**	**95% CI RDR**	***P***
*Performance*			
WHO 0	1		
1	1.3	1.0–1,8	0.069
2	2.4	1.6–3.5	<0.001
			
*Extent of disease*			
Locoregional	0.7	0.5–1.0	0.08
Limited dissemination	0.7	0.5–0.9	0.006
Extensive dissemination	1		
			
*LDH*			
Normal	1		
Elevated	1.5	1.1–2.0	0.021
			
*Paclitaxel*			
Yes	1		
No	1.3	1.1–1.7	0.015

RDR=relative death rate,

CI=confidence interval.

). WHO performance status and extent of disease are the strongest predictors for survival. Lactate dehydrogenase was a relevant risk factor, but was elevated in only 15% of patients. The type of treatment is an additional (external) factor determining prognosis.

**Figure 1:**
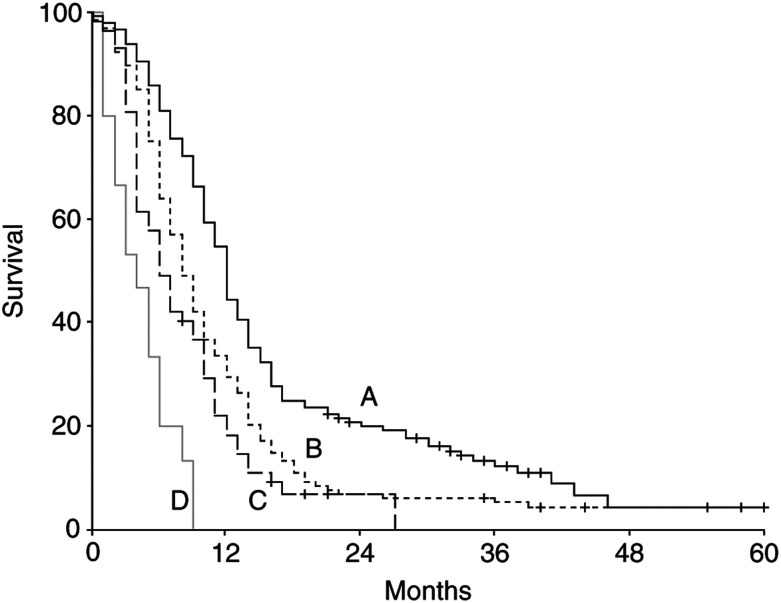
Kaplan–Meier survival curves for patients with 0–3 risk factors (A=no risk factor, B=one risk factor, C=two risk factors, D=three risk factors). The risk factors are WHO 2, extensive disseminated disease and elevated LDH.

We combined the patient characteristics such as performance status, extent of disease and LDH, to constitute four groups. A WHO performance score of 2, extensive disseminated disease and an elevated LDH were risk factors for poor survival, and the survival of patients with either 0, 1, 2 or 3 risk factors present was estimated. As shown in Figure 1

, there is a large difference in survival between these four patient groups. The median survivals of patients with 0, 1, 2 and 3 risk factors were 12, 8, 6 and 4 months, respectively. The 1-year survival rates were 45, 30, 18 and 0%, respectively. In the group of patients with three risk factors, no patient survived more than 9 months. Cox regression showed that the type of treatment did not significantly influence the relative differences in death rates between these four patient groups.

The relation between tumour response and survival was evaluated in patients who survived 4 months. Of the responding 137 patients, the median survival was 15 months as compared to 8 months of the 146 nonresponders (*P*<0.001). Cox regression showed that the response was a favourable prognostic factor, independent of the type of treatment and number of risk factors present. Adjusted for both these factors, the death rate among responding patients was reduced by 70% (*P*<0.001).

The characteristics of the group of patients with a survival of more than 3 years are listed in Table 5

**Table 5:** Patient characteristics of patients with a survival of 3 years (*n*=20)

**Characteristic**	**No. of patients**	**(%)**
*WHO performance status*		
0	10	50
1	8	40
2	2	10

*Extent of disease*		
Locoregional	5	25
Limited disseminated	12	60
Extensively disseminated	3	15

*Response to chemotherapy treatment*		
Complete response	4	20
Partial response	14	70
Stable disease	2	10

*Additional treatment after chemotherapy*		
Salvage surgery	13	65
Radiotherapy	4	20
None	3	15

. The majority of these patients had the following characteristics: a performance status 0 or 1, limited disease, partial or complete response to chemotherapy and additional treatment after chemotherapy with either radiotherapy or surgery. Out of 178 patients with locoregional disease or limited lymph node metastases, 60 patients received additional treatment, 31 patients received radiotherapy to a total dose of 50 Gy and 29 patients underwent an oesophageal resection. In all, 17 of these 60 patients (28%) survived at least 3 years.

## DISCUSSION

Knowledge of prognostic factors is essential for the management of individual patients and these factors should be taken into account in the design of randomised trails and in interpreting the results of such trials.

In patients with resectable oesophageal cancer, survival correlates closely with the extent of tumour infiltration in the oesophageal wall and the presence or absence of lymph node metastasis (Kato *et al*, 1993). Weight loss has also been identified as an independent prognostic factor in patients who were surgically treated with or without preoperative chemotherapy (Kelsen *et al*, 1998). Performance status has been reported to be an important prognostic factor in patients treated with radiotherapy alone (Coia *et al*, 2000). In a number of studies, the prognostic significance of biological factors such as oncogenes, tumour-suppressor genes and growth factors has been studied. Overexpression of p53, p21 and vascular endothelial growth factor has been identified as prognostic factors by some authors (Ikeda *et al*, 1999; Shimada *et al*, 2000), but the results of these studies are not always consistent and therefore these factors are currently not used in daily routine. Prognostic factor analyses in patients with locally advanced or metastatic oesophageal cancer treated with chemotherapy are scarce. Andreyev *et al* (1998) identified weight loss as an independent prognostic factor in patients with several gastrointestinal malignancies, but weight loss was not statistically significant in the subgroup of patients with advanced oesophageal cancer.

In this study, we analysed prechemotherapy characteristics in 350 patients with locally advanced or metastatic oesophageal cancer, who were treated with cisplatin-based chemotherapy. In multivariate analysis, performance status, extent of disease, LDH and the addition of paclitaxel to cisplatin were identified as the most important prognostic factors. Weight loss dropped out of the multivariate model when performance status was also included. This can be explained by the finding that most patients with a poor performance status also had significant weight loss. Metastatic involvement of the liver and the total number of metastatic sites lost their importance when studied with disease extent.

Based on the performance score, LDH and extent of disease, we were able to identify four prognostic groups. The median survivals of patients with zero, one, two and three risk factors were 12, 8, 6 and 4 months, respectively. Considering a median survival of 4 months for patients with three risk factors and the fact that none of these patients survived beyond 9 months, it is unlikely that these patients had any benefit from chemotherapy, at least with respect to survival. Tumour response as a post-treatment factor was found to be a favourable prognostic sign that was independent of the type of treatment and the number of risk factors present. However, the true merits of chemotherapy can only be assessed in randomised trials incorporating the stratification for risk factors and the measurement of quality of life.

Patients with lymph node metastases confined to either the celiac or supraclavicular lymph nodes had a better survival than patients with more extensive disease. The 3-year survival rate of the 136 patients with lymph node metastasis only was 11%. Most of the patients who survived more than 3 years after the start of chemotherapy were additionally treated with surgery or radiotherapy.

We found that patients treated in the more recent studies with dose-dense schedules of cisplatin and paclitaxel had a significantly better survival compared to patients treated with cisplatin and etoposide with or without 5-fluorouracil. The dose-dense schedules might bias the comparison of treatment schedules. Although the total dose of cisplatin was more or less comparable to the dose, the intensity (mg m^−2^ week^−1^) was significantly higher in the cisplatin and paclitaxel regimens. The improved outcome in the latter studies can be due to either the addition of paclitaxel or to the dose-dense character of these schedules.

In conclusion, performance score, extent of disease and LDH are independent prognostic factors in patients with advanced oesophageal cancer, who are treated with cisplatin-based combination chemotherapy. Patients with a poor performance, extensive disseminated disease and an elevated LDH have a poor outcome and should not be treated with cisplatin-based combination chemotherapy.
